# Vitamin D and Atopic Dermatitis in Childhood

**DOI:** 10.1155/2015/257879

**Published:** 2015-04-20

**Authors:** Michelangelo Vestita, Angela Filoni, Maurizio Congedo, Caterina Foti, Domenico Bonamonte

**Affiliations:** ^1^Section of Dermatology, Department of Biomedical Science and Human Oncology, University of Bari, 11 Piazza Giulio Cesare, 70124 Bari, Italy; ^2^Section of Dermatology, Vito Fazzi Hospital, Piazza Filippo Muratore, 73100 Lecce, Italy

## Abstract

Vitamin D features immunomodulatory effects on both the innate and adaptive immune systems, which may explain the growing evidence connecting vitamin D to allergic diseases. A wealth of studies describing a beneficial effect of vitamin D on atopic dermatitis (AD) prevalence and severity are known. However, observations linking high vitamin D levels to an increased risk of developing AD have also been published, effectively creating a controversy. In this paper, we review the existing literature on the association between AD and vitamin D levels, focusing on childhood. As of today, the role of vitamin D in AD is far from clear; additional studies are particularly needed in order to confirm the promising therapeutic role of vitamin D supplementation in childhood AD.

## 1. Introduction

Atopic dermatitis (AD) is a common chronic inflammatory condition characterized clinically by pruritus, eczematous lesions, and a defective epidermal barrier [1].

AD affects mainly children, but it may occur in adults [2]; these patients exhibit both impaired skin barrier function and defects in skin innate immunity [3].

AD is often associated with a personal or family history of type I allergies [4] (allergic rhinitis and asthma) and develops from a complex interplay between environmental, genetic, and immunologic factors.

Current treatment of severe disease is challenging because the safety profile of available systemic treatments limits its use.

## 2. Vitamin D

Vitamin D (also known as cholecalciferol) is an active steroid hormone. The function of vitamin D, traditionally associated with calcium and phosphate homeostasis, is thought to be highly complex, and its potential roles in cardiovascular, neoplastic, and microbial infections and autoimmune diseases have been investigated in recent studies [5]. Vitamin D deficiency and insufficiency in patients with asthma [6] and allergic diseases [7] have also recently been discussed.

Two independent pathways lead to vitamin D synthesis: the photochemical action of solar ultraviolet B (UVB) light in the skin and specific dietary sources. Vitamin D from supplements can be ingested as vitamin D2 from plant sources or vitamin D3 from animal sources [8].

Vitamin D3 is transported to the liver and is converted to 25-hydroxyvitamin D (25(OH)D). 25(OH)D is released into the circulation and is transported to the kidney where it undergoes further hydroxylation to 1,25-dihydroxyvitamin D (1,25(OH)D). This compound subsequently activates the vitamin D receptor (VDR), regulating the expression of genes involved in calcium metabolism, proliferation, differentiation, apoptosis, and adaptive immunity [9]. Individuals with higher phototypes, whose melanin functions as a natural sunblock, those who extensively use sunblocks, those who stay primarily indoors, and those living at high latitudes show a reduced vitamin D synthesis [10].

An inverse relationship between serum 25(OH)D concentration and age has been highlighted. The reason for this is presently unclear, although some have theorized that older children may take less oral supplementation or spend less time engaged in outdoors activity [11].

Factors such as obesity, gastrointestinal malabsorption, parathyroid hormone, calcium, phosphorus, fibroblast growth factor, and 1,25(OH)D itself can also alter 1,25(OH)D levels.

A 25(OH)D level of 20–30 ng/mL constitutes relative insufficiency and <20 ng/mL defines deficiency [12]. Data in adults suggests that vitamin D levels less than approximately 30 ng/mL are associated with changes in parathyroid hormone levels, as well as intestinal calcium transport [13]. Based on this, blood levels of 25(OH)D above 30 ng/mL should yield maximum health to the human body. As a matter of fact, vitamin D deficiency has become a public health issue. This has been largely attributed to dietary and lifestyle behaviors during recent decades [14]. Both children and pregnant/lactating women are identified as groups particularly vulnerable to vitamin D deficiency [15].

The American Academy of Pediatrics recommends a daily intake of 400 IU of vitamin D for infants and children, whereas the Institute of Medicine and The Endocrine Society recommend 400 IU daily for infants and 600 IU daily for children of 1 to 18 years old [16].

## 3. Vitamin D and Immune Regulation

The extent of vitamin D's biological actions goes further than calcium homeostasis and bone metabolism. Vitamin D features its immunomodulatory effects on both the innate and adaptive immune systems [17].

The VDR, a member of the nuclear receptor family [18], has been found on a wide range of immune cells, especially when activated, such as macrophages and T and B lymphocytes [19].

The activation of VDR on dendritic cells has been proven to modulate the tolerance of these antigen-presenting cells in adaptive immune responses. In detail Th2 cell response is enhanced not only by Th1 inhibition, but also as a result of balance shifting toward Th2 [20]; an effect on the differentiation of naive T cell into Th2 cells has been also noted [21].

Coherently to the above, Boonstra et al. [22] demonstrated that vitamin D inhibits IFN-*γ* production and promotes IL-4, IL-5, and IL-10 production in a mouse model.

These studies suggest that deficiencies in vitamin D levels and/or signaling would favor a predominant Th2 response and that the presence of vitamin D, while suppressing Th1 effects, also promotes Th2 responses. However limited data showed vitamin D might attenuate, rather than promote, Th2 responses.

## 4. Vitamin D and Skin Barrier Function

Vitamin D is also implicated into stratum corneum barrier formation, by means of protein synthesis (such as filaggrin) and regulation of keratinocytes proliferation and differentiation.

Vitamin D stimulates the production and the regulation of skin antimicrobial peptides, such as cathelicidins [23]. Among the hypothesized mechanisms, an action on the Toll-like receptor 2 seems to be primarily involved.

Antimicrobial peptides show both a direct antimicrobial activity and an induced host cellular response yielding to cytokine release, inflammation, and angiogenesis.

Given the above, vitamin D deficiency might predispose patients with AD to skin superinfection by* Staphylococcus aureus* or its superantigens [24].

## 5. Vitamin D Levels and AD Severity and Prevalence

Few studies have evaluated the prevalence and severity of AD in vitamin D deficient children.

Oren et al. [25], in a case-control study of 290 obese patients, showed that 5% of patients with vitamin D deficiency had AD, compared with 1% of the vitamin D-replete group. On the other hand no significant associations with asthma or allergic rhinitis were noted.

Peroni et al. [26] studied 37 children with AD with mild (*n* = 13), moderate (*n* = 15), or severe (*n* = 9) disease using the SCORAD index. They noticed that serum levels of 25(OH)D were higher in patients affected by mild AD compared to those with moderate or severe AD. Similar results were obtained by El Taieb et al. [27], who compared 29 AD children to a control group of 30 healthy individuals, and Wang et al. [28], who considered 498 Hong Kong Chinese children affected by AD and compared them to 328 controls. Overall these data seem to indicate that vitamin D deficiency is related to the severity of AD.

There are, however, many controversies. Despite the above evidences, several authors have had opposing results [29].

Bäck et al. [30] observed that higher intake of vitamin D during the first year of life was correlated with increased risk of eczema at age six. 123 children were investigated through a postal questionnaire looking for the cumulative incidence of AD, allergic rhinitis, or asthma at 6 years of age. Regardless of family history of atopy, AD was more prevalent in those with the highest intake of vitamin D.

Through a nationwide cross-sectional survey on 9838 German children and adolescents with eczema, Heimbeck et al. [31] found a significantly reduced risk of eczema for the lowest vitamin D serum quartile compared to the reference quartile in a multivariate analysis.

Chiu et al. [32] evaluated 94 children of 1 to 16 years old living in urban Milwaukee (USA), finding no statistically significant association between vitamin D levels and AD severity. Further, children with mild AD had serum levels of 25(OH)D lower than patients with moderate and severe diseases, although this difference was not statistically significant.

In conclusion, although there is a predominance of papers pointing to vitamin D as a protective factor, various studies actually identify it as a risk factor for AD. These controversial results could be explained by the hypothesis of Benson et al. [33], who proposed a bimodal and/or gender specific relationship between vitamin D and allergic skin diseases. Hyppönen et al. [34] also demonstrated a statistically significant nonlinear association between serum 25(OH)D and serum IgE that could account for this discrepancy. As a matter of fact, patients with low vitamin D (<10 ng/mL) or with very high vitamin D serum levels (>54 ng/mL) had significantly higher IgE levels than healthy individuals (40–50 ng/mL). Consequent correction of serum concentrations of vitamin D reduced IgE level significantly.

## 6. Climate Influence on AD and Vitamin D

It is well known that climate and sun/UVB exposure generally exert a beneficial effect on AD clinical course. As an example, a higher prevalence of AD in children who were born during autumn and winter was evidenced [35]. Similarly, AD usually worsens in winter as a consequence of reduced exposure to solar radiation and a higher prevalence of the dermatosis has been noted in countries at higher geographic latitude [36].

Since sun/UVB exposure increases vitamin D serum levels, some authors have logically hypothesized that AD clinical improvement by sun/UVB might be mediated at a molecular level by vitamin D.

This is supported by the observation that vitamin D deficiency is associated with more severe skin lesions localized on body sites that are not exposed to sun [37]. This would be a consequence of vitamin D decreased production in covered skin areas, thus indicating a local protective effect of vitamin D against the development of AD lesions.

Nevertheless, using five different population samples, Thyssen et al. [38] demonstrated that significantly higher concentrations of serum vitamin D were found in carriers of filaggrin mutations. This indirectly challenges the hypothesis that the increased prevalence of atopic disease can be a consequence of vitamin D insufficiency secondary to reduced solar/UVB exposure.

## 7. Association of Allergic Sensitization to AD and Vitamin D

Lee et al. [39], studying AD in 157 patients, 73.3% of which between the ages of 0–15, showed that, in the 36 patients with diagnosed food sensitization, mean serum levels of vitamin D were significantly higher in patients with mild AD (21.2 ± 5.18 ng/mL) compared to those with moderate (17.9 ± 4.02 ng/mL) or severe AD (13.3 ± 5.11 ng/mL).

Mohiuddin et al. [40] confirmed these results, further showing that, in patients with severe AD, for every 1-unit increase in serum 25(OH)D levels, the odds of food allergy development decreased by 6%.

Finally, assessing seventy-three children with AD, Akan et al. [41] demonstrated a negative correlation between the SCORAD [42] and serum vitamin D levels in allergic sensitized individuals, whereas no correlation was noted in the group without sensitization.

## 8. Vitamin D in Pregnant and Breastfeeding Women and AD

### 8.1. Pregnancy Vitamin D Levels

The maternal vitamin D profile during pregnancy has also been long debated. Camargo Jr. et al. [43] noted no decreased risk of AD in children whose mothers had higher intakes of vitamin D.

Gale et al. [44] reported that high vitamin D values during pregnancy might also be harmful with respect to allergic disease development: children whose mothers had a 25(OH)D concentration during pregnancy of greater than 30 ng/mL had an increased risk of atopic eczema on examination at 9 months compared with children whose mothers had a concentration of less than 12 ng/mL.

Conversely, other studies demonstrated that children born from mothers with low fish or vitamin D intake during pregnancy had an increased prevalence of AD [45, 46].

### 8.2. Cord Serum Vitamin D Levels

Finally, a significant inverse association was observed by Baïz et al. [47] between cord serum 25(OH)D levels and risk of transient early wheezing and AD by the ages of 1, 2, 3, and 5 years. Similar results were found by Jones et al. [48], who demonstrated that, for each 4 ng/mL increase in cord blood vitamin D levels, the risk of eczema diminished by 13.3%. From a pathogenetic perspective, the above observations are supported by evidence [49] indicating that low circulating 25(OH)D levels contribute to low IL-10 levels, the latter being notably associated with antiallergic proprieties. However there is also evidence to the contrary. Chi et al. [50] demonstrated an inverse association between cord plasma vitamin D levels and T-regulatory cells number.

### 8.3. Vitamin D Levels in Breastfeeding Women

There is evidence that breastfeeding in the first four months of life can reduce the risk of childhood eczema at 4 years of age [51].

Bäck et al. [30] also evidenced that breastfeeding is generally associated with low vitamin D intake, as opposed to replacement formulas and dairy drinks fortified with vitamin D, which provide a considerably higher intake.

Trying to elucidate whether maternal vitamin D supplementation during lactation improves infantile eczema and other subsequent allergic disorders, a randomized double-blind, placebo-controlled trial [52] was conducted on 164 breastfeeding mothers of infants with facial eczema.

The analysis showed that vitamin D supplementation may not decrease the severity of infantile eczema at 3 months of age but may rather increase the risk of later food allergy up to 2 years of age. The limits of this study include a large number of subjects lost at follow-up and the generic diagnosis of facial eczema (which comprises conditions other than AD).

Corroborating this association, Milner et al. [53] revealed that early infant multivitamin supplementation is associated with increased risk of food allergy and asthma in black ethnicity.

However, data on vitamin D dietary intake and higher prevalence of AD must be assessed critically.

Traditionally, food sensitization and higher incidence of atopy in children have been linked to increased intestinal permeability. Therefore, the increased prevalence of AD in children with a higher dietary intake of vitamin D could result from such early alimentary exposure to this antigen, rather than being a direct consequence of vitamin D serum levels.

## 9. Vitamin D Genes Polymorphisms

In 2002, Heine et al. [54] showed that, in adults with severe forms of AD, VDR gene polymorphisms were significantly overrepresented. This finding suggests that VDR may affect AD through regulation of epidermal barrier functions and cutaneous immune responses.

As a matter of fact, VDR can inhibit the maturation of dendritic cells and decrease proinflammatory cytokines such as IL-6 and TNF-*α*. Nonetheless, this haplotype also occurs with high frequency in the healthy population. Perhaps it acts more as a cofactor, which requires one or more environmental and genetic additional elements.

In 2014, Wang et al. [55] reported a genetic association study in which a vitamin D-related gene polymorphism rs4674343 on CYP27A1 was found to be protective against atopic eczema. Other genes (CYP2R1 and VDR) have been investigated and they may increase the susceptibility to develop eczema, as well as altering eosinophil percentage, and total IgE amount.

An interesting observation by van Belle et al. [56] demonstrated that certain polymorphisms in the VDR and metabolism genes might constitute genetic susceptibility factors for autoimmune diseases, although this requires further evidence to be confirmed.

There are also evidences to link the risk of atopy and asthma with polymorphisms in the VDR [57, 58].

## 10. Therapeutic Approach

### 10.1. Vitamin D Supplementation

A nutritional survey [59] comparing AD patients (*n* = 132) with healthy controls (*n* = 132) demonstrated that patients with AD had a lower vitamin D dietary intake than the control group. However, serum vitamin D levels were not measured.

Based on this rationale and comforted by the data obtained from observational studies, the following clinical trials investigated the therapeutic role of vitamin D supplementation in the treatment of AD.

In 2008 a double-blind randomized controlled trial in children with winter-related AD was performed [60] utilizing a regimen of 1,000 IU/day of vitamin D for one month during the winter. Five subjects received supplementation versus placebo in six subjects. Four of the five children who received vitamin D improved, whereas only one of the six children in the control group improved. The study was however limited by the small number of participants.

Other well-designed studies targeting exclusively a pediatric cohort are lacking; nonetheless a large amount of data is available on adult or mixed populations.

Javanbakht et al. [61] carried a randomized, double-blind, placebo-controlled study on forty-five patients with AD. Clinical improvement was assessed by SCORAD, which decreased significantly at 60 days in the groups receiving vitamin D or E or both vitamins.

A larger trial [62] also showed a significant reduction in SCORAD after vitamin D supplementation. 30 patients received vitamin D 1,600 IU/day and 30 patients received placebo. The treated group improved significantly at 60 days and had significantly higher serum vitamin D values than baseline, regardless of the initial severity of AD. In the placebo group, the improvement was not significant.

Furthermore, Hata et al. [63] tested a 3-week supplementation with 1000 IU/day vitamin D in 14 atopic subjects with moderate to severe AD, showing a significant increase in cathelicidins expression in lesional skin.

Mallbris et al. [64] confirmed this by showing that vitamin D leads to cathelicidins production and activation in keratinocytes. The above data may explain why skin infections occur more frequently in winter, when keratinocytes are less stimulated by vitamin D to produce antimicrobial peptides.

However, despite all of the above evidences, no significant difference in AD severity after vitamin D supplementation compared with placebo was found in a 2012 systematic literature review [65].

Trying to clarify the issue, in 2013, Samochocki et al. [66] carried out a study in which 20 out of 95 patients were selected for vitamin D supplementation (2000 IU of oral cholecalciferol daily); 25(OH)D mean concentrations were very low, between 4 and 15 ng/mL. After supplementation both mean objective SCORAD and SCORAD index were significantly lower than before. Similarly, after supplementation, all SCORAD parameters, except lichenification, were significantly decreased. After 3 months of supplementation most patients' vitamin D levels switched from <10 ng/mL to 10–20 ng/mL. For the whole supplemented group the subjective Patient Global Assessment parameter was between 0 and 3 points (mean 1.9). After 3 months of supplementation, the mean total IgE level was significantly lower than before.

As a corollary, in 2014, Borzutzky et al. [67] reported a case of vitamin D deficiency rickets in an adolescent with severe AD. Her serum 25(OH)D level was 4.8 ng/mL. Vitamin D supplementation increased her 25(OH)D level to 17.6 ng/mL, with normalization of alkaline phosphatase, parathyroid hormone, and calcium, as well as a noticeable improvement in her AD severity. This report, together with Samochocki et al.'s [66] observation, suggests that improvement may be more evident in case of severe vitamin D deficiency.

### 10.2. Heliotherapy

Different studies focused on the effect of heliotherapy on both vitamin D levels and AD severity. Vähävihu et al. [68] assessed 23 patients with AD from Nordic countries before and after daily heliotherapy in January (*n* = 11) or March (*n* = 12). Before heliotherapy, 17 of 23 patients had vitamin D deficiency; after 2 weeks of therapy, only 4 patients remained deficient. Of note, a positive correlation was evidenced between the increase in vitamin D levels and the decrease in the SCORAD index in March but not in January. The same authors carried out a later study [69] on 18 patients with AD. Of these, 16 were vitamin D deficient and underwent 15 sessions of narrowband UVB. This therapy resulted in a significant increase in serum vitamin D levels. Moreover, a significant decrease in mean SCORAD was recorded.

### 10.3. Topical Therapy

Certain observations further aimed at clarifying the role of topical vitamin D analogues. Evidence shows that topical application of the 1,25-dihydroxyvitamin D analogue is able to elicit an AD-like eruption in mice [70]. This reaction has been clarified to be not a simple irritant contact dermatitis, but rather a VDR- and thymic stromal lymphopoietin-dependent process [71, 72].

## 11. Conclusions

Epidemiological and clinical evidences indicate a beneficial role for vitamin D in AD. These observations are supported by basic research data showing that vitamin D acts on many different immune cell functions. However, how such a complex system may translate into nutritional guidelines and supplementation advice for the general population is yet to be understood.

Similarly, to devise a strategy for using vitamin D in the therapy of AD seems at present to be not feasible for different reasons: many confounding and unidentified variables appear in existing studies, and they are often limited by small numbers of participants, their short durations, and the use of a fixed dose without optimizing for adequate serum levels. Therefore systematic supplementation of vitamin D in childhood AD currently cannot be recommended except for uncommon cases which may prove refractory to traditional therapeutic options.

Additional studies, with adequate sample size, dose adjustment based on target serum vitamin D levels, longer duration of treatment, standardization of AD severity assessment, and adequate correction for confounding factors such as sun/UVB exposure and food intake, are currently much needed.
